# Phenylalanine as an effective stabilizer and aggregation inhibitor of *Bacillus amyloliquefaciens* alpha-amylase

**DOI:** 10.1186/s13568-024-01712-5

**Published:** 2024-06-08

**Authors:** Leila Adibi, Parichehreh Yaghmaei, Parvaneh Maghami, Azadeh Ebrahim-Habibi

**Affiliations:** 1 Department of Biology, Science and Research Branch, Islamic Azad University, North Sattaree Avenue, 1477893855 Tehran, Iran; 2 Biosensor Research Center, Endocrinology and Metabolism Molecular-Cellular Sciences Institute, Tehran University of Medical Sciences, Jalal-al-Ahmad Street, Chamran Highway, 1411713137 Tehran, Iran; 3 Endocrinology and Metabolism Research Center, Endocrinology and Metabolism Clinical Sciences Institute, Tehran University of Medical Sciences, 1411713137 Tehran, Iran

**Keywords:** Amino acids, Alpha-amylase, Amorphous aggregates

## Abstract

Aromatic compounds are known anti-amyloid aggregates. Their effect on amorphous aggregates of proteins is, however, less studied. We chose aromatic amino acids Trp, Tyr, and Phe, as well as another known stabilizer (i.e. Arg), as potential compatible solvents to be tested on *Bacillus amyloliquefaciens* alpha-amylase (BAA). Among these additives, Phe was the only one to be effective on the thermal inactivation and amorphous aggregation of BAA, while preserving its intrinsic activity. A concentration of 50 mM Phe was used to test its potential in counteracting the deleterious effect of BAA amorphous aggregates in vivo. After 21 days of daily subcutaneous injections of the native enzyme to mice, amorphous aggregates of BAA, as well as aggregates produced in presence of 50 mM Phe, the tissues located at the site of injection were studied histologically. Amorphous aggregates caused an increase in macrophages and lipid droplets. Serum levels of IL6 and TNF-α were also accordingly elevated and indicative of an inflammation state. Aggregates also resulted into increased levels of glucose, triglycerides and cholesterol, as well as liver enzymes SGOT and SGPT. On the other hand, the presence of Phe prevented this exacerbated inflammatory state and the subsequent impairment of biochemical parameters. In conclusion, Phe is an interesting compound for both stabilizing proteins and counteracting the pathological effect of amorphous aggregates.

**Supplementary Information:**

The online version contains supplementary material available at 10.1186/s13568-024-01712-5.

## Introduction

Proteins’ stability is a significant concern in the pharmaceutical industry, biochemical studies, and other biological investigations, since their native structure is sensitive to variations of environmental conditions (Manning et al. 2010). Physical instability of proteins results into denaturation, aggregation, precipitation, and adsorption, while chemical degradation occurs when covalent bonds are formed or cleaved, leading to the modification of their structures (Mojtabavi et al. 2019). These two process are interconnected in various systems (Manning et al. 2010).

A natural mean of stabilizing proteins in various organisms is the presence of osmolytes. These are usually small organic compounds such as amino acids, polyols, and methylamines. They indirectly impact the stability of macromolecules, by modifying the solvent properties in the cellular environment (Khan et al. 2010). The commonly acknowledged mechanism suggests that osmolytes are selectively kept away from the protein surface, initiating protein folding. This occurs due to a solvophobic interaction involving the peptide backbone, side chains present on the protein surface, and the “compatible” or “protective” osmolytes (Holthauzen et al. 2010). They can also affect protein aggregation by decreasing the accumulation of aggregation-prone and partially unfolded states, possibly by restoring the correct protein conformation (Khan et al. 2010; Mojtabavi et al. 2019). On the other hand, some osmolytes can interact with proteins backbone; and ultimately, the equilibrium between osmolyte–backbone interactions and amino acid side chain–solvent interactions determines the compatibility or incompatibility of the osmolyte and its impact on protein folding (Kumar 2009).

Concerning amino acids, stabilization studies have been performed especially with glycine, proline and arginine. Proline is suggested to be a “chemical chaperone” and has anti-aggregation properties at high concentration (Chattopadhyay et al. 2004), glycine acts generally as a stabilizer (Platts et al. 2015), while arginine shows mixed results which are a subject of various explanatory studies (Platts et al. 2015; Baynes et al. 2005; Arakawa et al. 2007; Kim et al. 2016).

The stabilizing or destabilizing properties of osmolytes may be generic and independent of a protein’s chemical characteristics, especially when considering the mechanisms that are proposed for their effects. However, some studies are indicative of different outcomes when using the same osmolyte for different proteins (Sharma et al. 2013).

Regarding phenylalanine, an earlier study indicated a neutral impact on protein stabilization. This was attributed to the offsetting of its dual effect: preferential hydration vs. favorable interactions occurring between the exposed hydrophobic side chains and the non-polar portion of the osmolytes (Taneja et al. 1994). On the other hand, our previous studies have shown a marked stabilizing effect of this amino acid on lysozyme stability and amorphous aggregation (Saadati-Eskandari et al. 2019).

Proteins stability and aggregation are interrelated. Amyloid aggregates and their deleterious effects on health have been the subjects of many studies, and anti-amyloid ligands have recently proven to be effective as drugs (Karran et al. 2022). However, there are still limited data on amorphous aggregates effects on health.

In this study, our objective was to test various amino acid additives on *Bacillus amyloliquefaciens* alpha-amylase (BAA) activity, stability, and amorphous aggregation propensity. Additionally, in continuation of our previous works, we aimed to investigate the anti-aggregation effect of the most promising additive in vivo.

## Materials and methods

### Materials and animals

Materials: Alpha-amylase from *Bacillus amyloliquefaciens* (BAA) was obtained from Sigma Aldrich (St. Louis, USA). Amino acids [arginine (Arg), tryptophan (Trp), tyrosine (Tyr), and phenylalanine (Phe)] were from Samchun Pure Chemical Co., Ltd. (Pyeongtaek, Korea) while other chemicals were from Merck. Ammonia content was measured with Ziestchem kit (Iran). Biochemical parameters were assayed with Pars Azmun kits (Iran), save for IL6 assay kit which was from MyBiosourse (USA), and TNF-alpha that was measured by Zellbio kits (Germany).

Animals: Thirty male NMRI mice with an average weight of 22 ± 2 g were purchased from the Pasteur Institute (Tehran, Iran) and kept in a controlled environment regarding temperature (22 ± 3 °C) and light conditions (12 h of darkness and 12 h of light). They had free access to food pellets and water.

### Enzyme activity assay

In order to measure alpha-amylase activity, Bernfeld’s method was used, which is a photometric end point assay based on dinitrosalicylic acid (DNS) reagent. Briefly, starch (substrate) is incubated with the enzyme for a specific duration and the reaction is stopped by adding DNS. DNS is also reacting with the product (i.e., maltose), creating a colored complex, which can be read by a spectrophotometer at 540 nm. The enzyme activity is measured as millimoles of maltose formed in 1 min. A standard curve of maltose concentration was also prepared beforehand (Bernfeld 1955).

### Thermal stability assessment

A suitable temperature to assess enzyme denaturation, where the enzyme’s residual activity approaches zero within a reasonable time period, was initially found to be 65 °C. Samples of the enzyme were placed in this temperature and incubated for 5–30 min. At regular intervals, samples were taken and put into ice for 30 min to prevent further potential denaturation of the enzyme. Residual activity percentage was then calculated using Bernfeld method and compared with the control sample, which had been on ice from the beginning of the experiment. The mean, standard deviation (SD), and coefficient of variation (CV) were calculated for each sample. CV value below 5%, was considered acceptable.

### Deamidation measurement

In order to determine ammonia content of samples, an enzymatic kit was used (ZiestChemDiagnostics, Tehran, Iran). The method is based on glutamate dehydrogenase (GDH) and the interconversion of alpha-ketoglutaric acid and glutamate, where alpha-ketoglutaric acid accepts a nitrogen atom from ammonia, and NADPH donates electrons in the process, getting oxidized to NADP+. The end products are glutamic acid (containing the nitrogen from the ammonia), and NADP+. The conversion of NADPH to NADP results into a decrease of absorbance AT 340 nm. The reaction rate depends solely on the concentration of ammonia and the activity of GDH (Van Anken and Schiphorst 1974). In silico prediction of deamidation propensities for glutamine (Q) and asparagine (N) were assessed with NGOME-Lite (Lorenzo et al. 2022).

### Amorphous aggregates formation

In order to investigate the optimal conditions for the formation of amorphous BAA aggregates, different concentrations of the enzyme were tested in KH2PO4 (100 mM with pH of 5 and 7, and 20 mM with pH 7) and at 65 °C. Formation of protein aggregates was monitored at 400 nm using turbidity (Haghighi-Poodeh et al. 2020) with a UV-Vis spectrophotometer equipped with temperature control (± 1) (Shimadzu TCC-240 A) and analyzed through photometric and kinetic measurements (using UVProbe software). Experiments were done in triplicates.

### Congo red assay and transmission electron microscopy (TEM)

To perform the test, and check for the presence of amyloid aggregates, 1000 µL of the Congo red (CR) buffer solution and 25 µL of the incubated and control alpha-amylase enzyme sample were mixed and left at room temperature for 10 min in order to stabilize the color. Absorption spectra of the sample was then recorded in a Shimadzu UV-1800 Spectrophotometer, at the wavelength range of 400 to 600 nm against the CR buffer as a blank. Stock solution of CR was prepared as follows: 7 mg/mL of CR was dissolved in a 0.005 M potassium phosphate buffer containing 0.15 mM NaCl (pH 7.4) and filtered through a 0.22 μm filter. TEM was performed with the use of a high resolution transmission electron microscopy (HRTEM, CM30, Philips, USA). Incubated BAA samples were applied to a Formvar carbon-coated grid Cu, Mesh 300. After eliminating extra liquid, the samples were stained by 2% (w/ v) phosphotungstic acid solution. The solution on the grid was allowed to dry for 2 min before being studied.

### Animals groups and treatment

After one week of acclimation to the new environment, the mice were randomly divided into five groups of six (*n* = 6). Groups were as follows:

-Control Group: Animals that did not receive any treatment during the experiment.
-Sham group: Animals that received a subcutaneous injection of 100 µL of solvent (KH2PO4 with pH 5) in the abdominal area.
-Experimental group 1 (BAA1): Animals that received a subcutaneous injection of 100 µL of a solution containing native (un-incubated) BAA with a concentration of 0.41 mg/mL in KH2PO4 solvent (pH 5) in the abdominal area.
-Experimental Group 2 (BAA2): Animals that received a subcutaneous injection of 100 µL of a solution containing amorphous aggregates of BAA with a concentration of 0.41 mg/mL in KH2PO4 solvent (pH 5) in the abdominal area.
-Experimental Group 3 (BAA3): Animals that received a subcutaneous injection of 100 µL of a solution containing amorphous aggregates of BAA with a concentration of 0.41 mg/mL in KH2PO4 solvent (pH 5) generated in the presence of 50 mM of Phe in the abdominal area.

Daily subcutaneous injections were administered in the abdominal area for a duration of 21 days (Saadati-Eskandari et al. 2022) after which the animals were subjected to a 12 h fasting. After being anesthetized, the animals were then euthanized by placement in a CO2 compartment. The present study was conducted in accordance with the National Institutes of Health guide for the care and use of Laboratory animals (NIH Publications No. 8023, revised 1978) and further approved by the Animal Ethics Committee of the Science and Research Branch at the Islamic Azad University (Tehran, Iran).

### Measured biochemical parameters

Mice serum samples were tested for glucose, triglycerides, cholesterol, liver enzymes alanine transaminase (ALT) and aspartate aminotransferase (AST) levels by photometric methods. The ALT test is based on its ability to catalyze the reaction of alanine and 2-oxoglutarate which results into glutamate and pyruvate. Pyruvate is then reacting with NADH in the presence of LDH (lactate dehydrogenase) to produce NAD + and lactate. The resulting NAD + production will decrease the absorbance of the solution at 340 nm. In the AST test, aspartate and 2-oxoglutarate react in the presence of AST and result into glutamate and oxoglutarate. Oxoglutarate will then react with NADH in the presence of MDH (malate dehydrogenase) to produce NAD + and malate. The resulting decrease in the absorbance of the solution at 340 nm upon NAD + production will be then measured. IL6 and TNFα were measured by the use of specific antibodies (enzyme-linked immunosorbent assay). Results are expressed as Mean ± SE. PRISM software was used to perform ANOVA and Tukey test. More specifically, one-way parametric ANOVA permitted comparison between groups and within groups. Tukey’s Multiple Comparison Test was used to determine the significant differences between the groups. In all tests, *p* < 0.05 was considered significant.

### Histological study

After 21 days of injections, palpable firm masses that were observed subcutaneously at the injection site in the experimental groups, were surgically excised. The excised tissue samples were fixed in a 10% formalin solution (pH 7) for 24 h. Then, routine laboratory procedures were carried out, including dehydration using various alcohol concentrations (70–100%), paraffin embedding, and sectioning with a microtome at 5 microns thickness. These sections were placed on slides and stained with hematoxylin-eosin staining, and Sudan black staining to detect fat tissue. After staining, different areas of the slides were magnified using a Labomed microscope (US) for photography and further analysis.

## Results

### Additives and thermal stability of BAA

In order to reach a suitable set-up for assessing the thermal stability of BAA, different temperatures were first utilized for BAA inactivation. Ultimately, we chose 65 °C (Fig. S1), since regular decrease of enzyme activity was achieved at a relatively short time.

Aromatic amino acids Trp, Tyr, and Phe, and the basic Arg were used as potential thermal stabilizer additives.

Using a range of concentrations (from millimoles to moles), we tried to find additives that were best able to alter the steep profile of BAA thermal inactivation in a dose-dependent manner, while doing it at minimum concentration. In this experiment, we have measured the percentage of the residual activity of BAA over time.

**Fig. 1:**
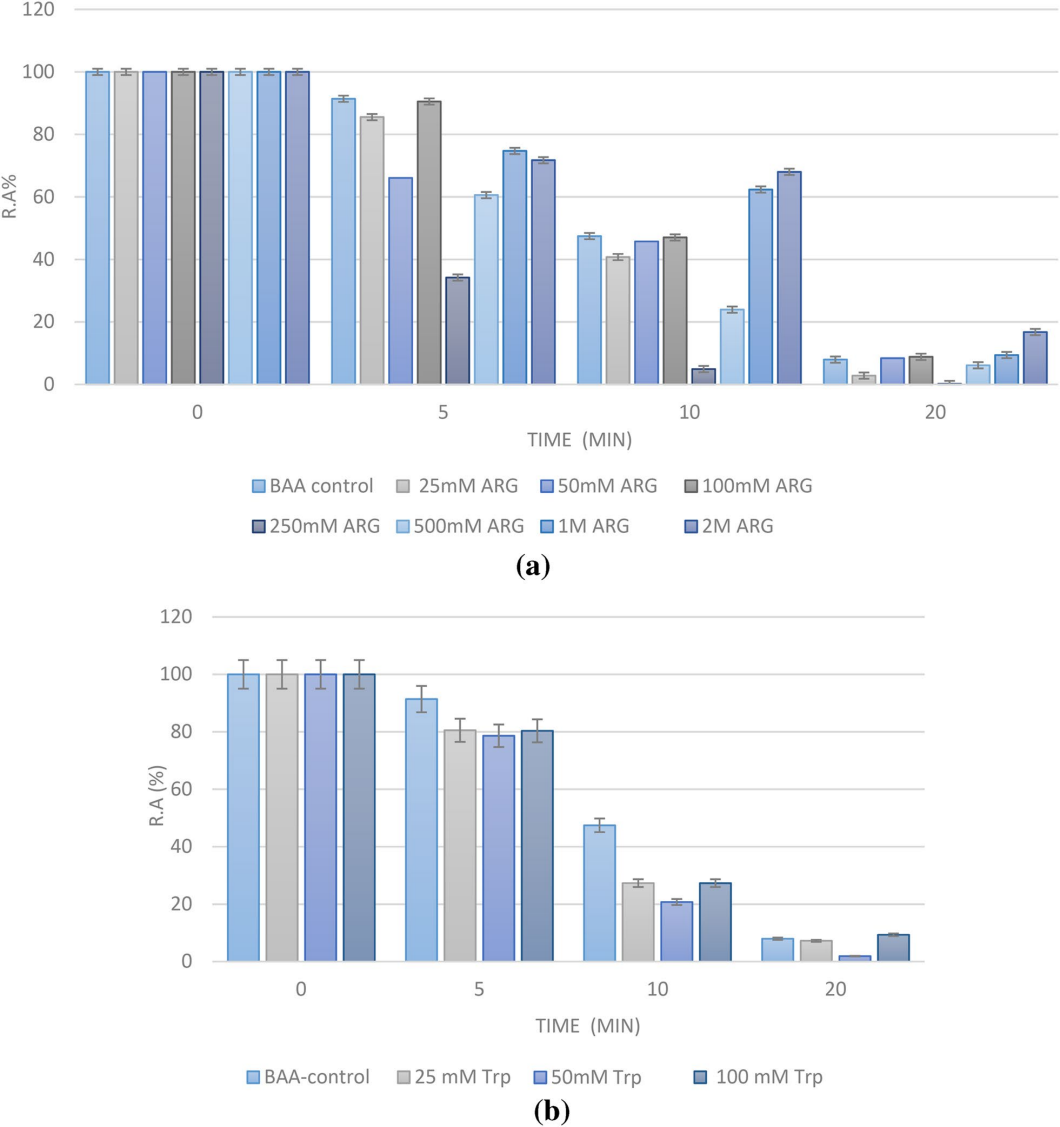

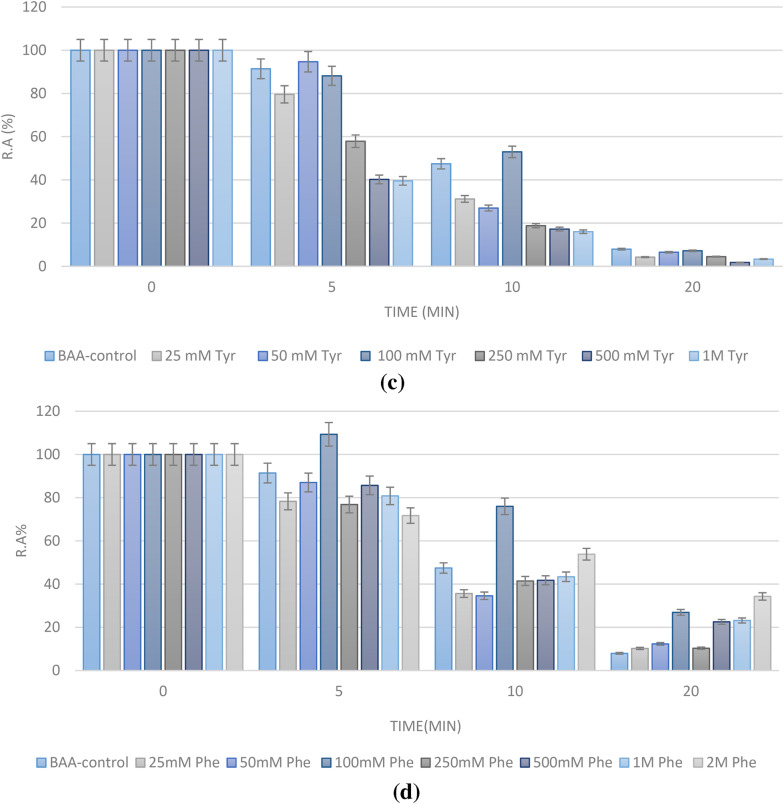
**a** Effect of various concentrations of Arg (25 mM-2 M) on BAA activity expressed as residual activity percentage (R.A%). **b** Effect of various concentrations of Trp (25–100 mM) on BAA activity expressed as residual activity percentage (R.A%). **c** Effect of various concentrations of Tyr (25mM -1 M) on BAA activity expressed as residual activity percentage (R.A%). **d** Effect of various concentrations of Phe (25mM -2 M) on BAA activity expressed as residual activity percentage (R.A%).

As shown in Fig. 1a, Arg is not able to prevent thermal inactivation over time, unless used at very high concentrations (higher than 500 mM, and especially at 2 M) as seen in the 10 and 20 min checkpoints. At 2 M, Arg is still able to keep around 16% of BAA activity (vs. 8% for the control enzyme) at the end. The problem with this high amount of Arg is that it affects the enzyme’s intrinsic activity. The reported percentages of Fig. 1a are relative to the first activity assessed for the enzyme in presence or absence of the additive. This means that although Arg at 2 M is able to maintain BAA activity, it does so in comparison to its first activity obtained in the presence of Arg (Table S1). As shown in Table S1, BAA activity remains intact in the presence of Arg up to 100 mM, after which it begins to decline. We considered adding other additives to Arg to balance its effect on BAA activity, but none of aspartate (Asp), glutamate (Glu), or lysine (Lys) could help (results not shown).

With Trp, the problem was its solvation in an aqueous buffer. Since we did not wish to add an organic solvent to the amino acids, the concentration range used for Trp was limited to 100 mM. As shown in Fig. 1b, the used concentrations were not able to counteract thermal denaturation of BAA, but seem to activate the enzyme to a little extent (Table S2).

When used at concentrations between 25 mM and 1 M, Tyr was also not effective as a BAA stabilizer (Fig. 1c). Even if it was the case, it could not be a suitable additive for BAA, since it has an even more pronounced effect on BAA activity compared to Arg. As shown in Table S3, the presence of Tyr at a concentration of 50 mM is already decreasing BAA activity to around 86%, while a sharp decline happens after this concentration reaches 100 mM (Table S3). The plotted Tyr concentration vs. activity percentage of BAA is shown in Fig. S2, and is suggestive of an inhibitory potential for Tyr.

Finally, Phe, which moderately affects BAA intrinsic activity (Table S4) was able to maintain BAA residual activity at the same level of the control sample in the initial period of thermal incubation, while it could preserve a higher level of activity in the latest segment, especially when used at higher concentrations (from 100 mM onward) (Fig. 1d).

As an additional experiment, we measured the amount of released ammonia in BAA environment over thermal incubation. This amount was 162.60 µg/dl for BAA alone and decreased to 9.56 µg /dl when BAA was incubated in presence of Phe (100 mM). BAA has 24 N residues and 23 Q residues. The sequence and predicted deamidation propensities for Q and N residues of BAA are also reported in Fig. S4.

Since Phe seemed to be a suitable candidate for further studies, we chose the 100 mM concentration, in order to have the least amount of additive that appeared to be effective as stabilizer.

### Phenylalanine and amorphous aggregates formation in BAA

**Fig. 2:**
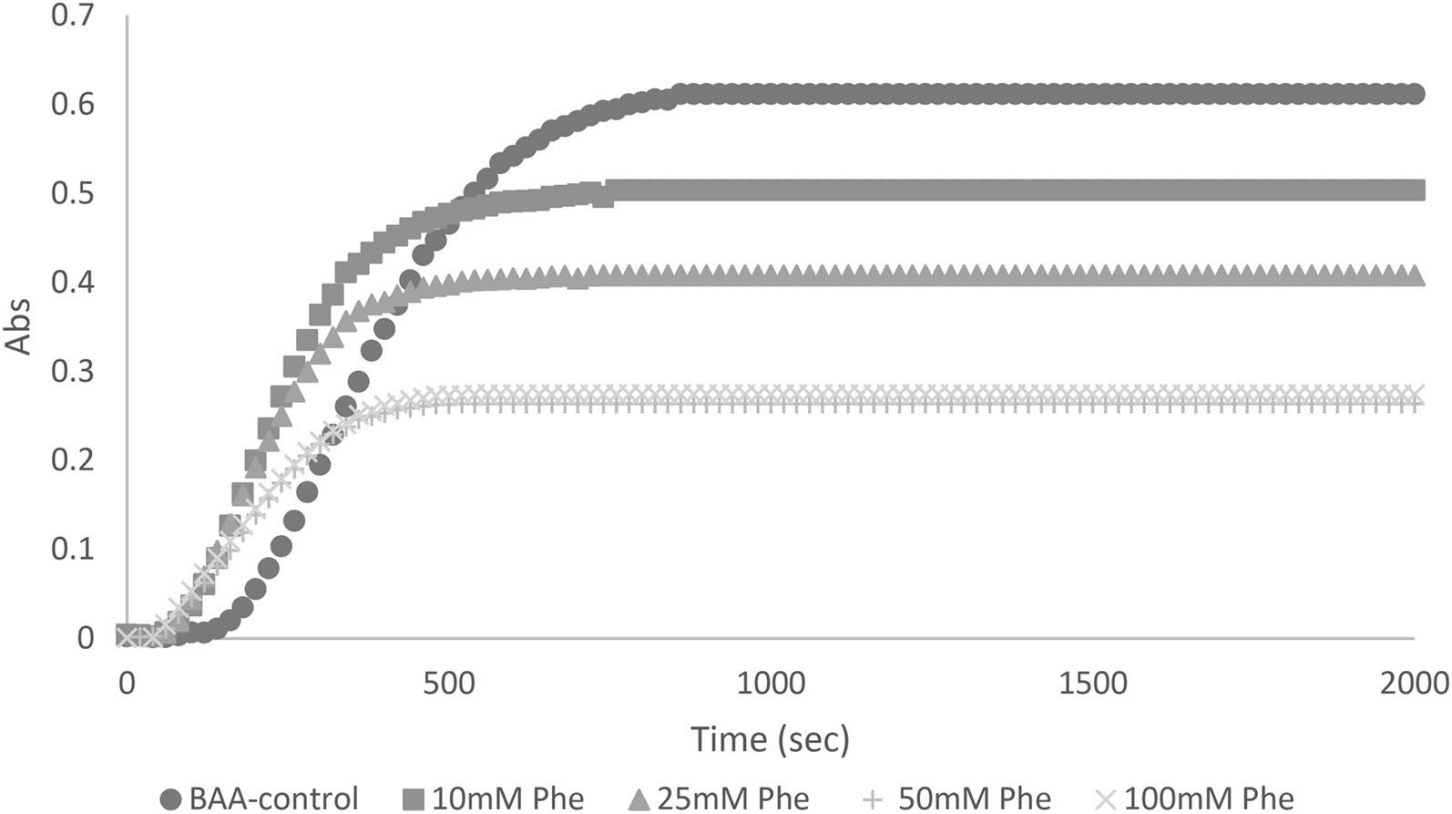
Aggregation plot of BAA (absorbance vs. time) in presence and absence of Phe (10–100 mM)

In the next step, we checked Phe ability to affect the formation of amorphous aggregates from BAA. As mentioned in the Methods section, the optimal conditions were first sought after, and we found out that aggregation would best occur at pH = 5. Absorbance spectra of BAA vs. time were then plotted. In these graphs, the maximal absorbance (plateau region on the plot) is indicative of an overall higher bulk of aggregates (Haghighi-Poodeh et al. 2020). As shown in Fig. 2, Phe at all concentrations used reduced the amount of aggregates in a dose-dependent manner, with a concentration of 50 mM performing best.

The next step was to confirm the amorphous nature of the produced aggregates. When Congo red dye is bound to amyloid structures, its absorbance spectrum is usually shifted with a change in intensity. This makes it a probe to detect amyloid aggregates (Buell et al. 2010). In our experiment, since the spectra of the dye alone, the protein alone, and the aggregated protein were almost identical, we may conclude that only amorphous aggregates were present in our samples (Fig. S3).

As a supplementary experiment, TEM experiment was performed to give a better visual proof for the amorphous nature of BAA aggregates. BAA aggregates were find to have typical disordered shapes (Fig. S5) similar to other previously detected amorphous species (e.g. Saadati-Eskandari et al. 2019).

### In vivo effects of BAA aggregates formed in presence and absence of phenylalanine: histological studies

There are scarce reports on the in vivo deleterious effects of amorphous aggregates. Actually, the most widely studied protein aggregates are the amyloid structures, which are related to pathologies such as Alzheimer’s and Parkinson’s diseases (Salahuddin et al. 2021). However, proteins’ amorphous aggregates can also be a cause of damage in the organs. In our previous works, we have started to assess the pathologic effect of various model proteins amorphous aggregates, alongside with investigation of aggregates attenuators on these processes (Saadati-Eskandari et al. 2022).

As reported in the Methods section, we have compared the effect of subcutaneous injection of native BAA, BAA aggregates, and BAA aggregates formed in the presence of phenylalanine. A subcutaneous injection of aggregates or amyloids for around three weeks has been shown to lead to the formation of a subcutaneous mass or at least an inflamed area (Azarfar et al. 2022) (Saadati-Eskandari et al. 2022). This mass is not formed in the absence of aggregates, i.e. with an injection of the native protein alone.

**Fig. 3:**
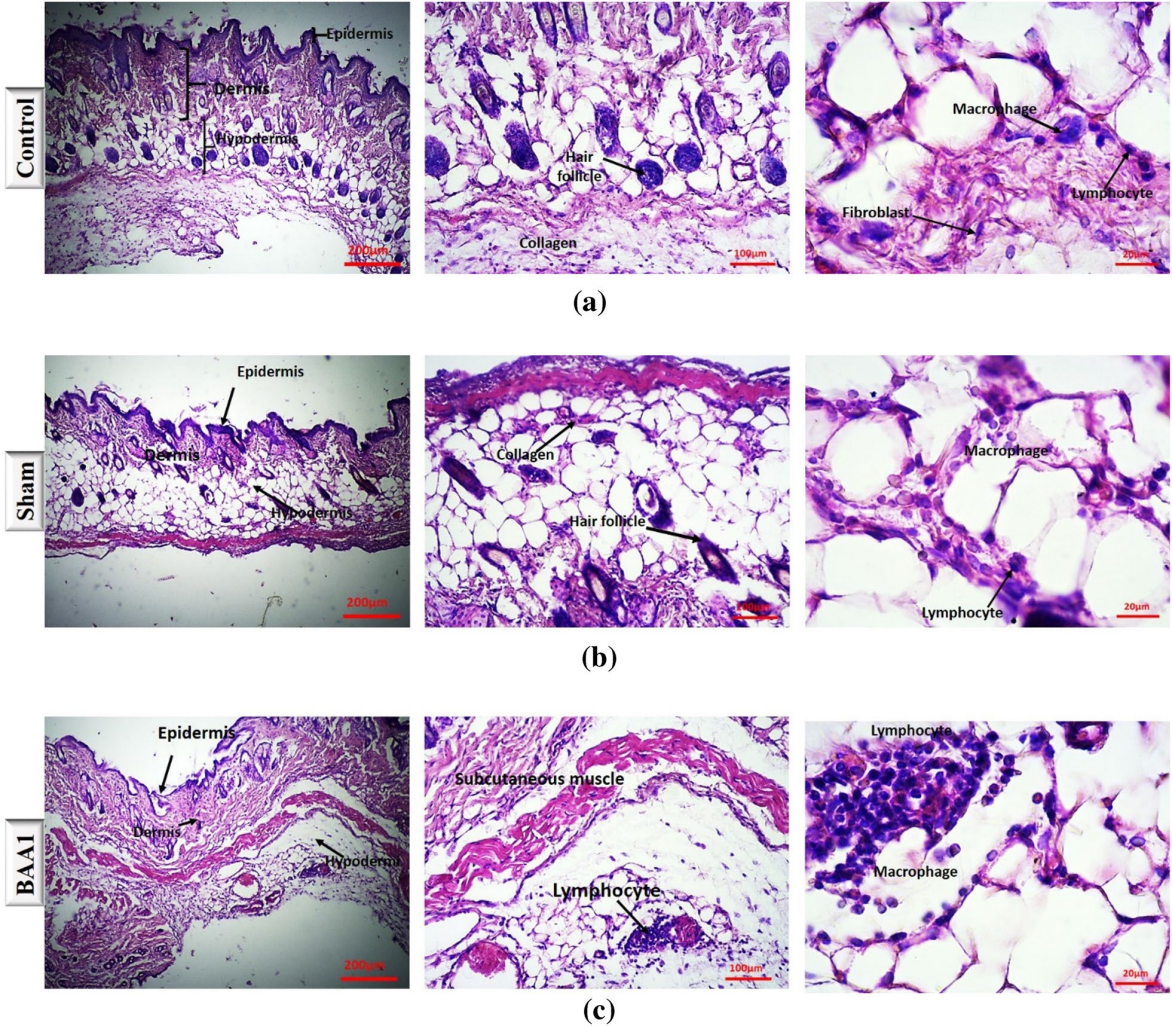

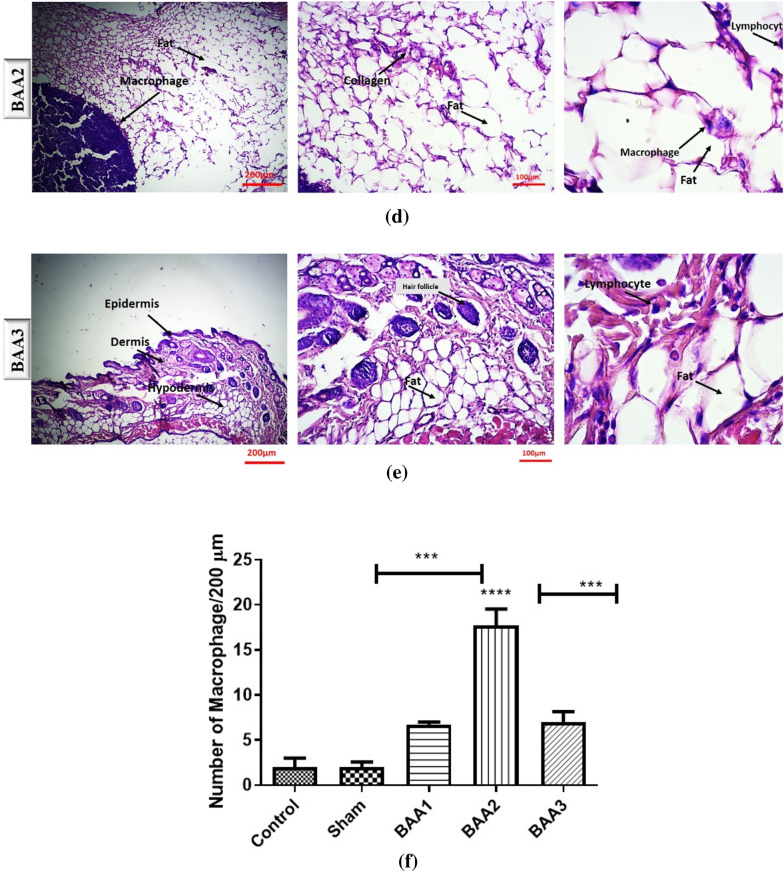
Results of H&E staining in control, sham, and the three experimental groups (**a**–**e**). **f** shows a statistical comparison between all groups macrophage numbers. *** *p* < 0.001, ****p < 0.0001

**Fig. 4:**
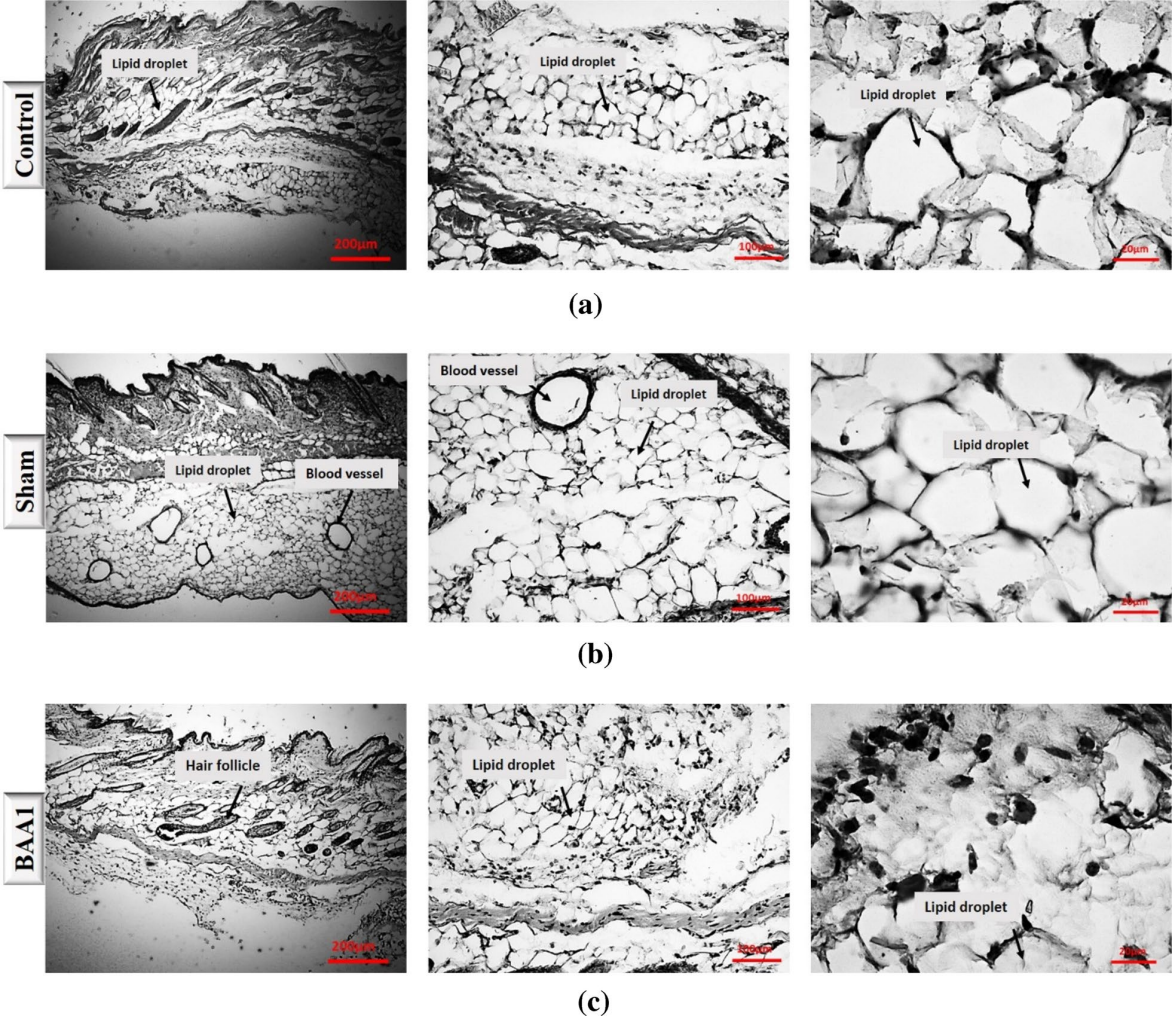

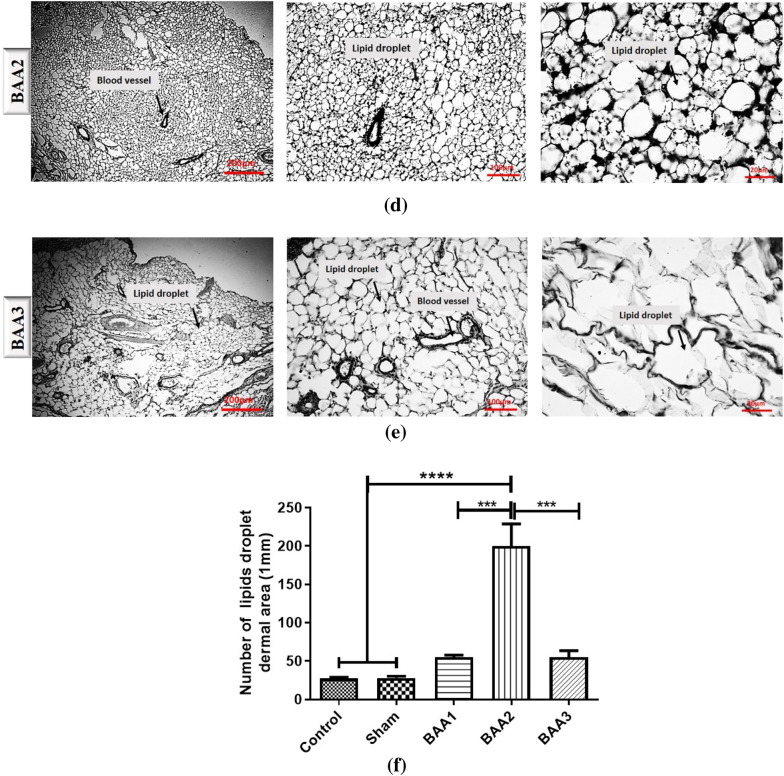
Results of Sudan black staining in control, sham, and the three experimental groups (**a–e**). **f** shows a statistical comparison between all groups lipid droplets numbers. *** *p* < 0.001, ****p < 0.0001

A comparative staining of the injected area was performed with hematoxylin and eosin (H&E) (Fig. 3a–f) as well as Sudan black (Fig. 4a–f).

The H&E images (Fig. 3) consist of cross-sectional views of mouse skin tissue with the epidermis, dermis, and hypodermis, as well as fibroblastic cells and differently arranged collagen bundles. In this staining, cell nuclei appear purple, while cell cytoplasm is pink. In addition, inflammatory cells, including lymphocytes and neutrophils with rounded and multi-lobed nuclei were observed. A quantified analysis was performed on the number of macrophages, which could be an indication of the inflammatory state of a tissue (Oishi et al. 2018).

As shown in Fig. 3f, in the control and sham (injection stress) groups, the population of these cells was very low and considered normal. This is in contrast with the BAA2 group (aggregated protein), where the population of macrophage cells responsible for phagocytosis was very high. In BAA3 (aggregates formed in presence of Phe) and BAA1 (native protein), this population was lower, with no significant difference between the two groups. In summary, in a 200-micrometer area the number of macrophage cells in the BAA1 study group was approximately 6, while being 17 in the BAA2 group, and 7 in the BAA3 group. One-way parametric ANOVA (Analysis of Variance) with a significant level of P value < 0.0001 was used for comparisons between and within groups.

The Sudan dyes have a wide range of applications for lipid staining in the microstructures of organisms and Sudan Black B is widely used for lipid staining in tissues. With Sudan Black, we found that the highest values of lipid accumulation were observed in the BAA2 group. There was a significant difference in terms of lipid droplet accumulation between the control group and the BAA2 group (Fig. 4). However, no significant difference was observed between the control group and both BAA1 and BAA3. A significant difference in terms of lipid accumulation values was observed between the Sham and the BAA2 group, but no significant difference was observed with the other two groups (BAA1 and BAA3). Other analyses showed that there was a significant difference in terms of lipid accumulation values between the BAA1 and BAA3 groups compared to the BAA2 group, but no significant difference was observed between the BAA1 and BAA3 groups (Fig. 4f).

### In vivo effects of BAA aggregates formed in presence and absence of phenylalanine: biochemical parameters

**Fig. 5:**
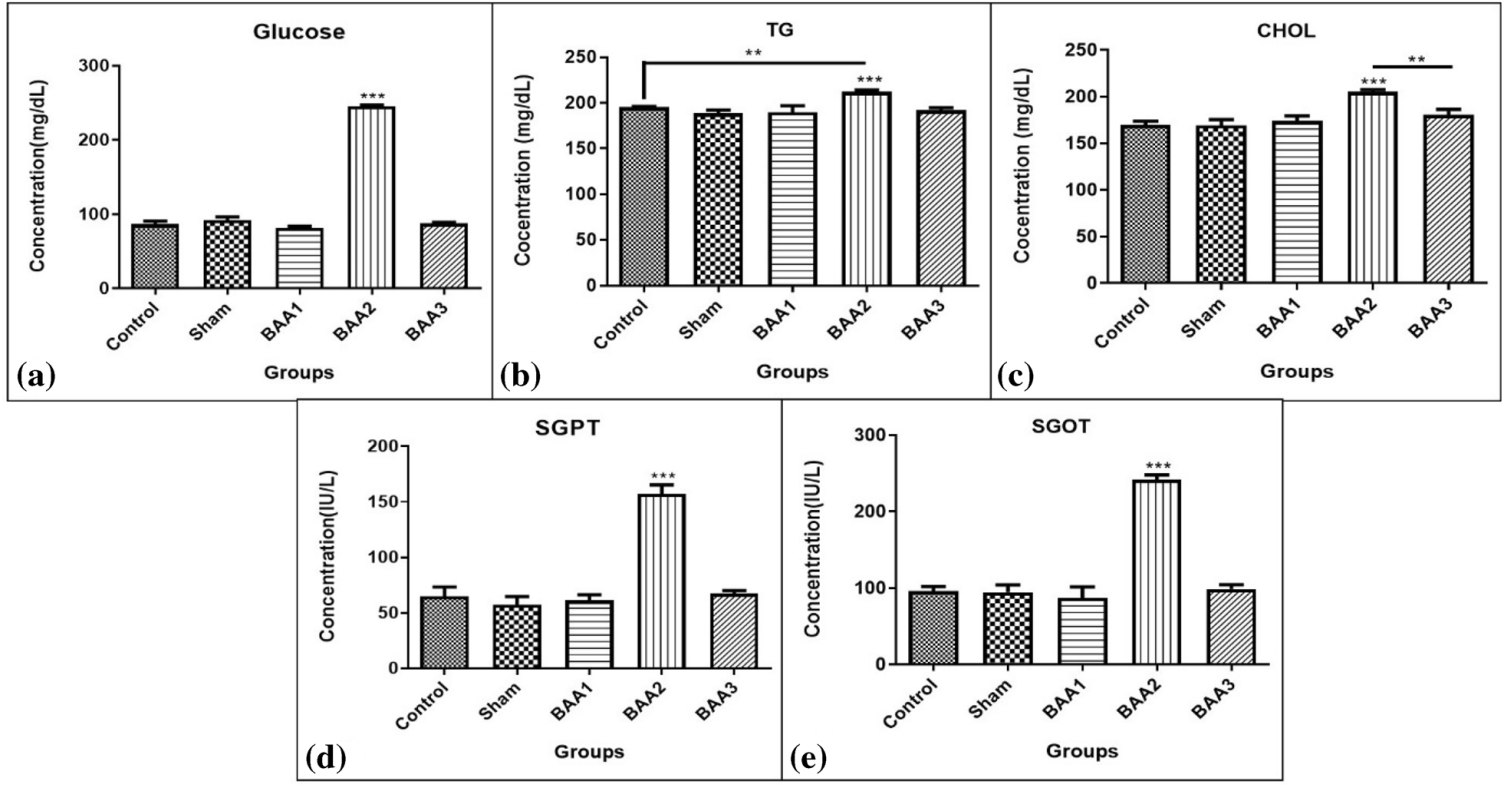
Comparison of fasting blood glucose, triglycerides (TG), cholesterol (CHOl), SGPT and SGOT serum levels between control, sham, and experimental groups. **p < 0.01, ***p < 0.001, ****p < 0.0001

Biochemical parameters, i.e., fasting blood glucose, cholesterol, triglycerides, as well as SGPT and SGOt (hepatic enzymes) levels were investigated in the serum of control, sham, and experimental groups (Fig. 5).

Glucose levels averages were 81.89 mg/dL in the BAA1 group, 245.3 mg/dL in the BAA2 group, and 87.55 mg/dL in the BAA3 group. Cholesterol levels averages were 174 mg/dL in the BAA1 group, 205 mg/dL in the BAA2 group, and 180.3 mg/dL in the BAA3 group. Triglyceride levels average were 189.7 mg/dL in the BAA1 group, 212 mg/dL in the BAA2 group, and 192 mg/dL in the BAA3 group.

Statistical analysis showed that there was a significant difference between the control group and the BAA2 group for all these parameters, but no significant difference was observed between the control and other groups. The Sham and the BAA2 groups showed also a significant difference, but no significant difference was observed with the other two groups (BAA1 and BAA3). Intra-group comparison showed a significant difference in triglyceride levels between the BAA1 and BAA2 groups, but no significant difference was found between the BAA1 and BAA3 groups. A significant difference was also observed between the BAA2 and BAA3 groups. P levels were different, though, depending to the biochemical parameter that was measured, and the glucose levels of BAA2 were remarkably increased (Fig. 5).

Average levels of SGOT were 87.33 IU/L in the BAA1 group, 242 IU/L in the BAA2 group, and 98.67 IU/L in the BAA3 group, while average levels of SGPT were 61 IU/L in the BAA1 group, 157 IU/L in the BAA2 group, and 67.33 IU/L in the BAA3 group. These results show a difference between all groups and BAA2 while no significant difference was found between the other groups themselves (Fig. 5).

### Phenylalanine and BAA amorphous aggregates in vivo: cytokine changes

**Fig. 6:**
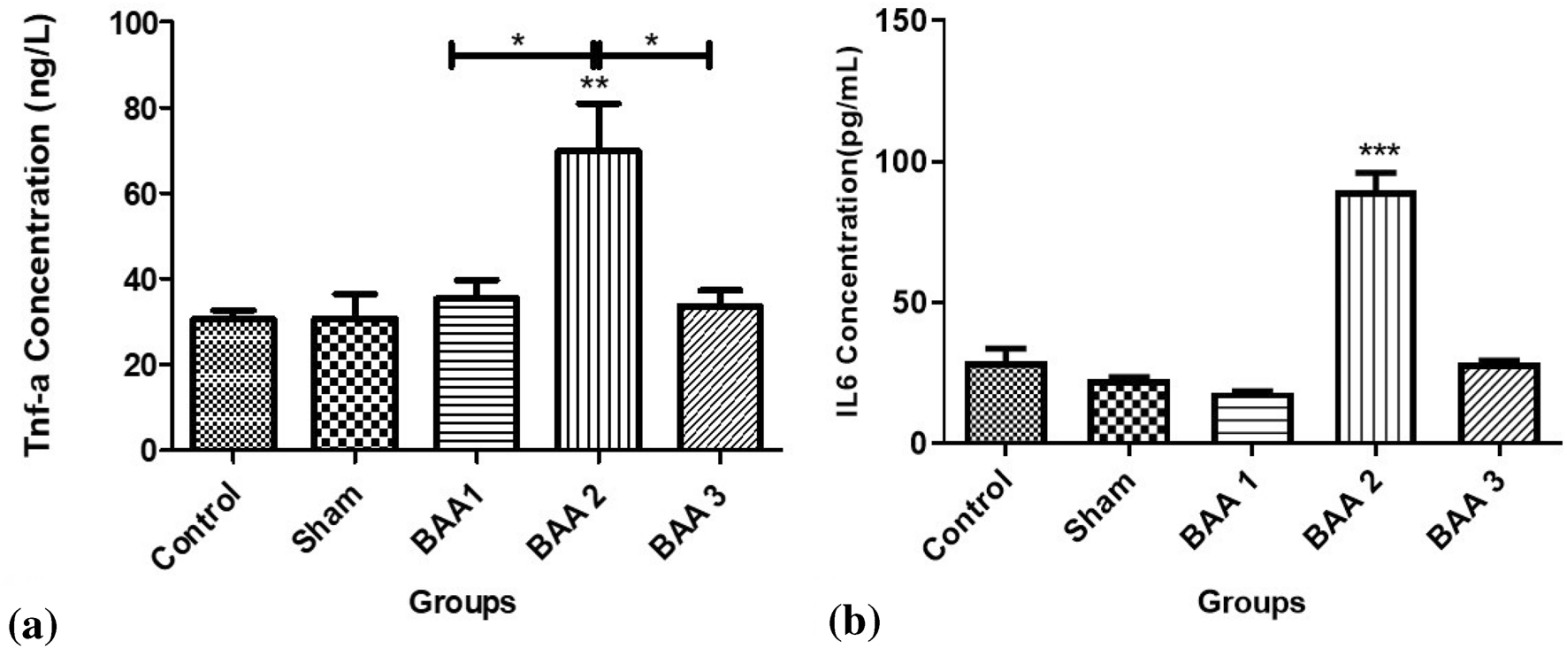
TNF-α (**A**) and interleukin 6 (**B**) concentrations compared in the different groups. *p < 0.05, **p < 0.01, ***p < 0.001

Serum levels of Tumor necrosis factor alpha (TNFα) and Interleukin-6 (IL-6) were also investigated and compared (Fig. 6). TNF-α average level was 35.48 ng/L in the BAA1 group, 69.85 ng/L in the BAA2 group, and 33.73 ng/L in the BAA3 group, while IL-6 levels were 18.11 pg/mL in the BAA1 group, 89.81 pg/mL in the BAA2 group, and 28.51 pg/mL in the BAA3 group.

Statistical analysis showed that a significant difference could be observed between BAA2 and the other groups, while for BAA1 and BAA3, there was no significant difference between them or with control and sham groups (Fig. 6).

## Discussion

Achieving stabilization of proteins, alongside with diminishing their aggregation propensity would, on one hand, elongate their shelf-lives, and on the other hand, prevent or decrease potential harmful effect of aggregates.

Using compatible solutes, i.e., molecules that can protect proteins and do not interfere with their structures and functions is a convenient way to stabilize therapeutic or industrial proteins. While some amino acids and carbohydrates are already known and used in this regard, there is still room to find other effective molecules (Bhojane et al. 2022).

Our choice of potentially stabilizing additives Arg, Trp, Tyr, and Phe, was based on previous reports in the literature, and our own studies on other proteins (Baynes et al. 2005; Saadati-Eskandari et al. 2019, 2022; Haghighi-Poodeh et al. 2020). These additives effect was investigated on BAA, which is one type of alpha-amylase, an enzyme that could be considered a therapeutic target (Kaur et al. 2021), and an industrial enzyme (Souza 2010). As a therapeutic target, it is inhibited to decrease post-prandial glucose in patients with diabetes, and as an industrial enzyme, among its several uses, it acts as a detergent aid.

Arg could be a stabilizer or destabilize proteins (Kim et al. 2016). Regarding BAA, while effective at maintaining a percentage of the enzyme’s activity when used at high concentration, it affects its intrinsic activity as an inhibitor. The inhibitory property manifests itself at concentrations higher than 100 mM, which does not make it a specific inhibitor for BAA. Nevertheless, Arg cannot be considered as an effective stabilizer of BAA. Adding Glu to Arg had been found effective in enhancing Arg stabilization ability, and a balance in the charges (or ions) could also be one reason for this effect. Both the conformational and colloidal stability of proteins could be positively affected by this combination (Golovanov et al. 2004; Kheddo et al. 2014). However, as mentioned in the Results section, neither adding aspartate (Asp) nor glutamate (Glu) could influence Arg effect on BAA. We also tried lysine (Lys), since it was an effective stabilizer in another set of experiment on a different alpha-amylase (unpublished results), but it was also ineffective and we discarded Arg from the potential stabilizers.

Trp, Tyr and Phe have all aromatic components, which are known inhibitors of amyloid aggregates formation. In general, indole (found in Trp) and phenol or phenyl groups (found in Tyr and Phe), are all reported as effective in amyloid structure formation inhibition in various proteins e.g. (Jayamani et al. 2014; Mohammadi et al. 2016; Goyal et al. 2018; Chaari 2020). Since both amyloid and amorphous aggregates may derivate from a common oligomeric intermediate (Almeida et al. 2020), it is possible that some of their initial structures may be inhibited by similar small molecules.

Concerning BAA, Trp could not stabilize the protein with the used concentration, and Tyr was found to be an inhibitor with an approximate IC50 of 100 mM. While this is also not very specific as inhibitory property, Tyr structure could be considered in order to design inhibitors of BAA.

Phe, on the other hand, was able to stabilize BAA to some extent, while not being too harsh on its activity. We further continued our tests with Phe, and found that it was also able to suppress BAA amorphous aggregation. Our previous experiments with lysozyme had shown a marked effect for Phe in the conformational and colloidal stability of the enzyme. Phe may interact with protein residues via pi-pi and cation-pi interactions, forming stacking stabilizing interactions as well as hydrophobic interactions that may preserve the hydrophobic patches from interacting with each other, and aggregate (Saadati-Eskandari et al. 2019). It has also been demonstrated that Phe residues that are present in the structure of proteins may have a stabilizing role by interacting with Glu and Asp residues via anion-pi interactions (Philip et al. 2011). Furthermore, as demonstrated by measuring released ammonia in BAA environment, Phe is also able to decrease deamidation, that occurs especially in asparagine and glutamine, and is one of the covalent changes happening to proteins upon exposition to high temperatures (Khajeh et al. 2001). The highest propensity for deamidation is likely to occur at the termini of BAA (Q2 and Q512), as well as N149, N152, N154, and N356 (Fig. S4). Protein termini are probably more readily exposed to deamidation, as they may be more water accessible. Concerning the other cases, since deamidation is started with a nucleophilic attack from water molecules, the presence of residues with side chains that withdraw electrons or stabilize positive charges of N or Q carbonyls may accentuate deamidation. For example, N149 is preceded by E147, while N152 is followed by R153, which has a guanidium group apt to stabilize the positive charge of the carbonyl. N154 is in the same location, and followed by Q155 which is also relatively prone to deamidation (Fig. S4). Q155 is followed by E156. Thus, the 147–156 segment seems to be a particularly vulnerable region. Finally, N356 is preceded by E355 and followed by H357 which may be both helping the process. Additives such as Phe may directly interact with these weak points, or neighbor Glu residues.

In the next step, we verified whether Phe was able to counteract the possible in vivo harmful effect of BAA amorphous aggregates. Subcutaneous injections of the amyloid-prone peptide insulin result in the formation of palpable masses in patients (Nagase et al. 2014). We have previously replicated this process in animals, and found out a marked inflammatory state both at the injection site and at serum level (Azarfar et al. 2022). Our initial experiments involved injecting amorphous aggregates of human lysozyme, which led to the formation of a less conspicuous mass at the injection site (Saadati-Eskandari et al. 2022). With regard to BAA, we found palpable masses over a 21 days injection, and impaired glucose, lipid, and liver enzymes profile in the animals. Interestingly, we had previously observed a marked change in fasting blood glucose levels of animals injected with lysozyme aggregates, while the presence of Phe had been able to prevent this change (Saadati-Eskandari et al. 2022).

The substantial increase in the cytokine levels TNFα and IL-6 over aggregates injection is consistent with an inflammatory state that is discernible at the histological level, where a significant increase happened in the number of macrophages and lipid droplets. Both these cytokines are pro-inflammatory factors and may be produced by macrophages (Shapouri-Moghaddam et al. 2018), as well as the adipose tissue (Kern et al. 2001).

It has been demonstrated that TNFα elevation in chronic inflammatory states may be related to impaired lipid profiles and higher risk of cardiovascular diseases (Sorokin et al. 2022). Both these cytokines are also linked with insulin resistance, and thus, higher levels of glucose. The role of TNFα has been well-defined and is agreed upon (Hotamisligil et al. 1993). For IL-6, this effect is manifested when it is chronically elevated and provokes inflammation, while in acute situations, it may actually have “insulin-like effects” (Lee et al. 2018).

SGPT and SGOT levels may increase as a result of liver damage, such as a state of steatohepatosis, over the consumption of substances that cause hepatotoxicity, or in cases of hepatitis (liver inflammation). They also play a role in gluconeogenesis by transferring amino groups from aspartic acid or alanine to ketoglutaric acid, forming oxaloacetic acid and pyruvic acid. Higher levels of SGPT indicate hepatocyte damage, and not necessarily cell death. Both SGPT and SGOT correlate with obesity (Lala et al. 2022), elevated blood glucose levels, insulin resistance, and, overall, metabolic syndrome (Hanley et al. 2005). Furthermore, systemic inflammation is associated with these markers of liver function (Browning et al. 2008).

As observed in our previous test with lysozyme (Saadati-Eskandari et al. 2022), as well as a recent study on a different amylase enzyme (unpublished results), amorphous aggregates, when present for three weeks in the body, will definitively cause an inflammatory state that is not limited to their location. As mentioned above, this state manifests itself at serum level, and influences other factors, such as liver damage markers, glucose levels, and lipid profile. Further studies are required to better elucidate the correlation between these factors and the potential impact of amorphous aggregates on other biochemical parameters.

In conclusion, phenylalanine appears to be a promising compatible solute for use as a protein stabilizer. Based on our results, its effect is hypothesized to be a generic one, but our experiments have been limited, and there is a need to test this compound on other, different proteins. An important property of Phe is its ability to protect against the harmful manifestations of proteins’ amorphous aggregates, which is a great potential for a putative additive in therapeutics.

### Electronic supplementary material

Below is the link to the electronic supplementary material.

**Supplementary Information**

## Data Availability

All data are available within the manuscript and supplementary materials. The raw data are available upon reasonable request from the corresponding authors.
