# Supplementary material for: Phenylalanine as an effective stabilizer and aggregation inhibitor of Bacillus amyloliquefaciens alpha-amylase
Source: AMB Express. 2024 Jun 8;14:69. doi: 10.1186/s13568-024-01712-5 (PMC11162409; doi:10.1186/s13568-024-01712-5)
Supplement: Supplementary file 1 — Supplementary Information [file 13568_2024_1712_MOESM1_ESM.docx]

**Supplementary Information**

**Fig. S1 :** Testing 60 (⚫) and 65 °C (⚫)for the thermal stability experiment of BAA . R.A %: Remained activity percentage.

Fig. S2. Effect of Tyr (25 mM-2M) on BAA activity.

Fig.S3 Congo red experiment: assessing BAA control, aggregates and Congo red alone spectra.


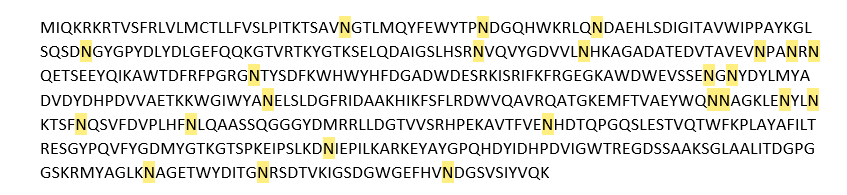


N:24 occurrences


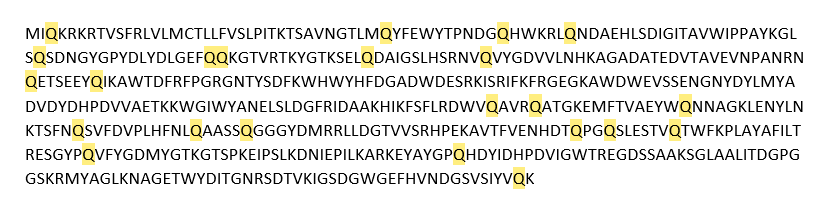


Q: 23 occurrences


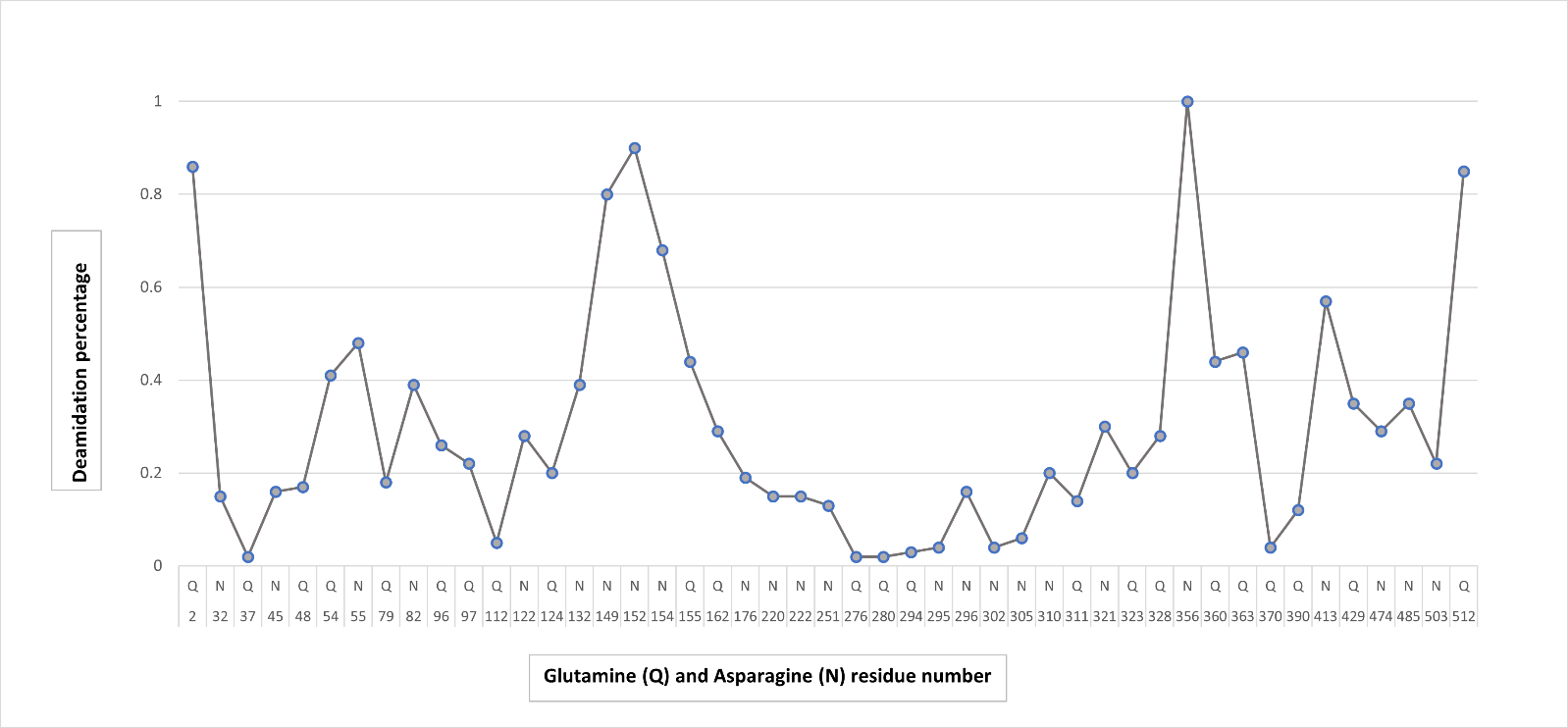


Figure S4. BAA sequence with N and Q highlighted. BAA has 513 residues. The plot shows NGOME assessment of

deamidation propensities for N and Q residues in BAA.


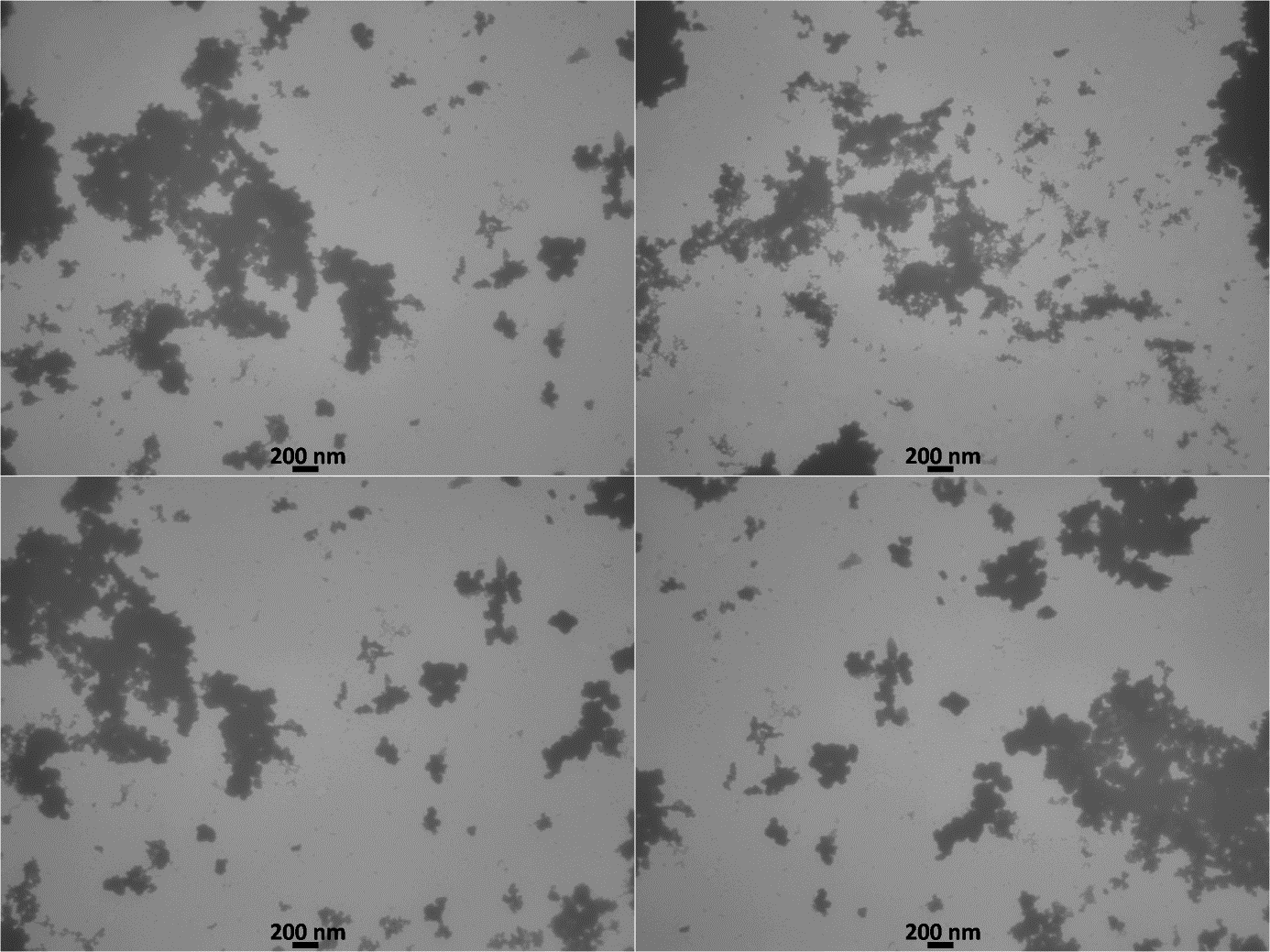


Fig.S5 TEM images of aggregates formed by BAA.

**Table S1** Effect of Arg concentrations on BAA activity

| Arg Concentration | BAA Activity (%) | |
| --- | --- | --- |
| 0mM | 100% |  |
| 25mM | 109,80% |  |
| 50 mM | 155,43% |  |
| 100 mM | 94,49% |  |
| 250 mM | 30,02% |  |
| 500 mM | 20,05% |  |
| 1000 mm | 2,11% |  |
| 2000mM | 1,33% |  |

**Table S2** Effect of Trp concentrations on BAA activity

| Trp Concentration | BAA Activity (%) | |
| --- | --- | --- |
| 0mM | 100% |  |
| 25mM | 99,93 |  |
| 50 mM | 103,20 |  |
| 100 mM | 120,90 |  |

**Table S3** Effect of Tyr concentrations on BAA activity

| Tyr Concentration | BAA Activity (%) | |
| --- | --- | --- |
| 0mM | 100% |  |
| 25mM | 94,61% |  |
| 50 mM | 85,93% |  |
| 100 mM | 56,13% |  |
| 250 mM | 7,25% |  |
| 500 mM | 7,23% |  |
| 1000 mm | 2,35% |  |

**Table S4** Effect of Phe concentrations on BAA activity

| Phe Concentration | BAA Activity (%) | |
| --- | --- | --- |
| 0mM | 100% |  |
| 25mM | 98,82% |  |
| 50 mM | 81,56% |  |
| 100 mM | 69,60% |  |
| 250 mM | 91,87% |  |
| 500 mM | 83,47% |  |
| 1000 mm | 101,22% |  |
| 2000mM | 78,99% |  |
